# Supplementary material for: Effects of Sodium Arsenite on the Myocardial Differentiation in Mouse Embryonic Bodies
Source: Toxics. 2023 Feb 1;11(2):142. doi: 10.3390/toxics11020142 (PMC9965385; doi:10.3390/toxics11020142)
Supplement: Supplementary file 1 [file toxics-11-00142-s001.zip › toxics-2163032-supplementary.pdf]

Effects of sodium arsenite on the myocardial differentiation in mouse embryonic stem cells

Supplemental Table S1. qPCR primer information

Pathway	Gene	Forward	Reverse	Ref
Pluripotency	*Oct*	TCAGCTTGGGCTAGAGAAGG	TGACGGGAACAGAGGGAAAG	(Cao et al., 2012)
	Sox2	GGCGGCAACCAGAAGAACAG	GTTGCTCCAGCCGTTCATGTG	
	Nanog	TCTCCTCGCCCTTCCTCT	ATGCGTTCACCAGATAGCC	
	c-Myc	ATGCCCCTCAACGTGAACTTC	CGCAACATAGGATGGAGAGCA	
	Klf4	GTGCCCCGACTAACCGTTG	GTCGTTGAACTCCTCGGTCT	
	Rex1	AGCAGGATCGCCTCACTG	GGCCACTTGTCTTTGCCG	
Cardiac function regulator and transcription	*Nppa*	GCTTCCAGGCCATATTGGAG	GGGGGCATGACCTCATCTT	
	Gja1	ACAGCTGTTGAGTCAGCTTG	GAGAGATGGGGAAGGACTTGT	
	Mef2c	GATGCAGACGATTCAGTAGG	TGGTGCCTGCACCGGATGTC	
	Isl1	TGTCAGGAGACTTGCCACTTT	GCCAAACGTTTATTAGTGAAATAGT	
Cardiac muscle	*Myl2*	AAAGAGGCTCCAGGTCCAAT	CCTCTCTGCTTGCGTGGTTA	
	Myl7	CCCATCAACTTCACCGTCTTCCT	AGAGAACTTGTCTGCCTGGGTCA	
	Myh6	GATGCCCAGATGGCTGACTT	GGTCAGCATGGCCATGTCCT	
	Tnnt2	GCGGAAGAGTGGGAAGAGACA	CCACAGCTCCTTGGCCTTCT	

	Myh11	AAGCTGCGGCTAGAGGTCA	CCCTCCCTTTGATGGCTGAG	
	Mkl2	ATGCCTTGAGGGAAGCAACC	GCTCGCTCCAGGCTTTTTATC	
CA^{2+} control and gap junction	*Slc8a1*	AGGTCCATGCTAGAGATCATCC	CATCATCGTCATCTTCCCCA	
	Cacna1a	CCTGCTGGTGGTTAGCGTG	TCTGCCTCCGTCTGTTTGGAA	
	Ryr2	ACATCATGTTTTACCGCCTGAG	TTTGTGGTTATTGAACTCTGGCT	
Hematopoietic	*Gata1*	TGGGGACCTCAGAACCCTTG	GGCTGCATTTGGGGAAGTG	
	Cbfa2t3	CCACGGCTGCTTAAAGTGGT	GTCATTGCCAAATTGCTGTAGG	
Endothelial	*Pecam1*	GTCATGGCCATGGTCGAGTA	CTCCTCGGCATCTTGCTGAA	
	Cdh5	CACTGCTTTGGGAGCCTTC	GGGGCAGCGATTCATTTTTCT	
Endodermal	*Sox17*	GCCAAAGACGAACGCAAGCGGT	TCATGCGCTTCACCTGCTTG	
	Afp	GCTCAGCGAGGAGAAATGG	CTTCACCAGGTTAATGAGAAGC	
Ectodermal	*Sox1*	AATCCCCTCTCAGACGGTG	TTGATGCATTTTGGGGGTAT	
	Nestin	AGAGAAGCGCTGGAACAGAG	AGGTGTCTGCAACCGAGAGT	
Differentiation	*Brachyury*	TATGAGCCTCGAATCCACATAGT	CCTCGTTCTGATAAGCAGTCAC	(Lee et al., 2021)
	Tbx20	AAGAAAGACCACACGGCCTC	GTTCTTCAAAGGTCCCCTCTCA	
	Tnni3	AGCCACACGCCAAGAAAAAGT	CGTGAAGCTGTCGGCATAAG	
	GATA4	AGGGAGGGGATTCAAACCAG	CTGCTGTGCCCATAGTGAGA	(Ko et al., 2006)
	Ncx1	AGGGGAAGACTTTGAGGACA	GAAGCCACCAAGCTCATTCA	
Apoptosis	*Bcl-2*	TAAGCTGTCACAGAGGGGCT	TGAAGAGTTCCTCCACCACC	(Kang et al.,

	Bax	CGAGCTGATCAGAACCATCA	GAAAAATGCCTTTCCCCTTC	2017)
	Caspase3	GATAATGTCTTAGAACTTGAATCC	CTTCCATAAATCAGGTCCAA	
ER stress	*Atf4*	TCGATGCTCTGTTTCGAATG	AAGCAGCAGAGTCAGGCTTC	
	CHOP	CCTGAGGAGAGAGTGTTC	CTCCTGCAGATCCTCATAC	
	BiP	CTATTCCTGCGTCGGTGTGT	GCAAGAACTTGATGTCCTGCT	
Mitophagy	*Parkin*	TCCGAAGATTCCTACCTTCC	AGGGGCTGCTTCTGTAATCT	
	PINK1	GCCAACACTGAACTTTGCTT	GCTGGTTGCTGCTTACAAAT	
	Duox1	GCTGAGAAGTTCGACCTCAG	CAGACTCCTGTTCAGCACCT	
Oxidation	*SOD2*	GAGTTGCTGGAGGCTATCAA	CGACCTTGCTCCTTATTGAA	
	GSR	AGCTGTGAGGGTAAATTCAG	AGCTGTGAGGGTAAATTCAG	
	Gss	AAAAGGACGACTATACTGCC	TGTAATCTGAGCGATTCAGG	
	Nox3	CTGGGATGAAAGTCTGGATG	GGGTGATTGTAGGCAATCTG	
Mitochondria	*Complex I*	TTCTTGCAGCTGTGTCCAAC	AGCATTTTGGGAGGGTTCTT	(Jung et al., 2022)
	Complex II	ACACAGACCTGGTGGAGACC	GGATGGGCTTGGAGTAATCA	
	Complex III	TGGTCTCCCAGTTTGTTTCC	GCAGCTTCCTGGTCAATCTC	
	Complex IV	TGCTCAACGTGTTCCTCAAG	TAAGGGTCCAAAACCAGTGC	
	Complex V	CGGACAGATGTCCTTCACCT	ACTTAGTCGTGGTGCCGTCT	
Internal control	*18S*	CTCAACACGGGAAACCTCAC	CGCTCCACCAACTAAGAACG	
	CalbindinD-9K	TAAAGACTATAAAAGAGCCCCTC	CTGGGGAACTCTGACTGAAT	

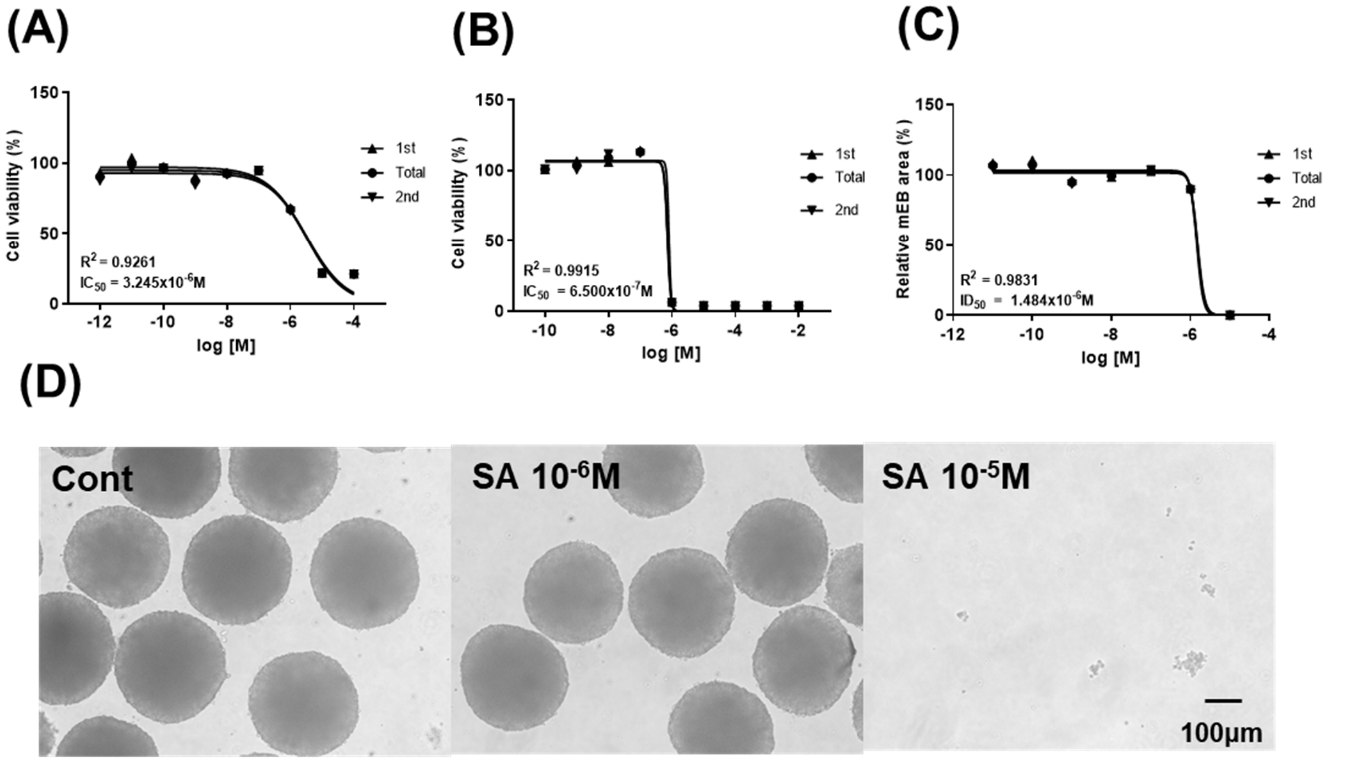

Supplemental Figure S1. Developmental toxicity classification of sodium arsenite. The mESCs and 3T3-L1 cell cytotoxicity assay. The IC_{50} value was measured using a CCk-8 assay, and the ID_{50} was detected by measuring the radius of EB. After the SA treatment for four days (A) Dose-response curve of the cell viability in mESCs with SA. (B) Dose-response curve of cell viability in the 3T3-L1 cells with SA. (C) Dose-response curve of relative mouse embryonic body (mEB) area with SA and more than 40 EBs were measured for each four group. (D) Representative image of each group on the optical microscope. Scale bar = 100 μm. Each value is expressed as means ± SD. n = 6 (A and B), Cont; control, SA; sodium arsenite.

Supplemental Table S2. Endpoint values of sodium arsenite on EBT.

Endpoint	Cell type	Value [μM]	Value* [Log_{10}]	R square (R^2)
IC_{50}	mESCs	3.245	0.511	0.9261
	3T3-L1	0.650	-0.187	0.9915
ID_{50}	EBs	1.484	0.171	0.9831
**SDF / Classification		2.593/ Toxicant		

*log10 value was calculated using μM value.

**SDF: Score of discriminant function

References

Cao, N., Liu, Z., Chen, Z., Wang, J., Chen, T., Zhao, X., Ma, Y., Qin, L., Kang, J., Wei, B., Wang, L., Jin, Y., & Yang, H.-T. (2012). Ascorbic acid enhances the cardiac differentiation of induced pluripotent stem cells through promoting the proliferation of cardiac progenitor cells. *Cell Research*, *22*(1), 219-236. https://doi.org/10.1038/cr.2011.195

Jung, E. M., Yoo, Y. M., Lee, J. H., & Jeung, E. B. (2022). Cytotoxicity evaluation and mechanism of endocrine-disrupting chemicals by the embryoid body test. *Toxicological Research*. https://doi.org/10.1007/s43188-022-00132-6

Kang, M. H., Das, J., Gurunathan, S., Park, H. W., Song, H., Park, C., & Kim, J. H. (2017). The cytotoxic effects of dimethyl sulfoxide in mouse preimplantation embryos: a mechanistic study. *Theranostics*, *7*(19), 4735-4752. https://doi.org/10.7150/thno.21662

Ko, I., Lee, C. H., Lee, K. P., Lee, S. W., & Kim, K. W. (2006). Remediation of soil contaminated with arsenic, zinc, and nickel by pilot-scale soil washing. *Environmental Progress*, *25*(1), 39-48. https://doi.org/10.1002/ep.10101

Lee, J. H., Yoo, Y. M., Lee, B., Jeong, S., Tran, D. N., & Jeung, E. B. (2021). Melatonin mitigates the adverse effect of hypoxia during myocardial differentiation in mouse embryonic stem cells. *J Vet Sci*, *22*(4), e54. https://doi.org/10.4142/jvs.2021.22.e54
